# The case of value-based healthcare for people living with complex long-term conditions

**DOI:** 10.1186/s12913-016-1957-6

**Published:** 2017-01-11

**Authors:** Marie Elf, Maria Flink, Marie Nilsson, Malin Tistad, Lena von Koch, Charlotte Ytterberg

**Affiliations:** 1 School of Education, Health and Social Studies, Dalarna University, Falun, Sweden; 2 Department of Neurobiology, Care Sciences and Society, Karolinska Institutet, Huddinge, Stockholm, Sweden; 3 Department of Social Work, Karolinska University Hospital Huddinge, Stockholm, Sweden; 4 Department of Neurobiology, Karolinska University Hospital Huddinge, Stockholm, Sweden; 5 Functional Area Occupational Therapy & Physiotherapy, Karolinska University Hospital Huddinge, Stockholm, Sweden

**Keywords:** Policy healthcare, Value-based care, Health expectations, Patient-centred care, Stroke, Rehabilitation

## Abstract

**Background:**

There is a trend towards value-based health service, striving to cut costs while generating value for the patient. The overall objective comprises higher-quality health services and improved patient safety and cost efficiency. The approach could align with patient-centred care, as it entails a focus on the patient’s experience of her or his entire cycle of care, including the use of well-defined outcome measurements. Challenges arise when the approach is applied to health services for people living with long-term complex conditions that require support from various healthcare services. The aim of this work is to critically discuss the value-based approach and its implications for patients with long-term complex conditions. Two cases from clinical practice and research form the foundation for our reasoning, illustrating several challenges regarding value-based health services for people living with long-term complex conditions.

**Discussion:**

Achieving value-based health services that provide the health outcomes that matter to patients and providing greater patient-centredness will place increased demands on the healthcare system. Patients and their informal caregivers must be included in the development and establishment of outcome measures. The outcome measures must be standardized to allow evaluation of specific conditions at an aggregated level, but they must also be sensitive enough to capture each patient’s individual needs and goals. Healthcare systems that strive to establish value-based services must collaborate beyond the organizational boundaries to create clear patient trajectories in order to avoid fragmentation.

**Summary:**

The shift towards value-based health services has the potential to align healthcare-service delivery with patient-centred care if serious efforts to take the patient’s perspective into account are made. This is especially challenging in fragmented healthcare systems and for patients with long-term- and multi-setting-care needs.

## Introduction

This paper discusses the value-based approach—i.e. the relation between the organization, performance and payment of a health services and its achieved outcome—and its implications for patients living with long-term conditions and complex needs, i.e. conditions that require long-term support from various healthcare professionals in various health services. For this group of people we have identified some challenges that we argue needs to be addressed when applying value-based health services. Our points of views are based on our multi-professional clinical experience and research projects in Sweden, which focus on health in everyday life for people with long-term complex conditions. Our research group is well established national and international and involves persons with background in nursing, social work, rehabilitation and physiotherapy. The overarching question of this paper is how health service should ascertain that value-based care is based on patient perspective. We will present two cases based on narratives from persons with stroke and health service data from our research to illustrate some of the challenges and research questions that we propose needs to be further addressed.

## Background

The call for health services based on the patient’s and family’s needs and expectations has been part of the international agenda in recent decades. The term patient-centred care implies that the care should be based on the patient’s perspective and goals and on shared decision-making [1, 2]; this approach is regarded as a key quality factor in contemporary healthcare [3]. Patient-centred care is prominent in legislation and policy documents around the world, and Swedish healthcare legislation has recently been revised to further strengthen patients’ rights [4] and much work remains to be done before health services can be considered fully patient-centred [5]. Swedish Agency for Health and Care Services Analysis [6, 7] has produced several reports that shows that Sweden needs to improve patient-centred care also in comparison with other countries.

There is currently a trend to move health services towards value-based organization, striving for a service that cuts costs while generating value for the patient [8, 9]. For example, the Karolinska University Hospital in Stockholm—the largest hospital in Sweden—is currently transforming the healthcare delivery to a value-based health service. The overall objective with value-based health services is to achieve higher-quality health services alongside improved patient safety and cost efficiency. The approach could align with patient-centred care if it focuses on the patient’s experience of her or his entire cycle of care, including the use of well-defined outcome measurements [8, 9], as opposed to older health-service models designed to focus on individual service activities and interventions.

### Value-based health service

Porter [9], one of the initiators of value-based care, defines value in terms of the “health outcomes achieved per dollar spent.” Thus, value-based care constitutes health services that create added value by focusing on how the service is organized, performed and paid for in relation to the outcomes achieved in terms of, for instance, patients’ health and experiences of health services. Important in value-based healthcare is the transition towards reimbursement for patients’ entire care cycle rather than for a visit to a single care provider. A cycle of care can contain several episodes; although it might be possible to delineate the cycle in the context of a surgical procedure, as described by Porter [8], doing so is more challenging in the context of a complex long-term health condition. Moreover, a core element in value-based healthcare constitutes measures of quality. Health outcomes are to be measured from the patient’s perspective and their support networks (e.g., families) rather than using process measures [8] and should, when it comes to long-term complex conditions, include measurements of the patient’s maintained functioning [10]. Lilford and colleagues [11] contrast outcome—and process measures and state that both are needed to monitor quality in healthcare. Process outcomes should be used to monitor the quality of clinical practice or process.

The value-based healthcare approach is a promising development, although it involves challenges in the area of health services for people living with long-term complex conditions. The potential problems highlighted in this paper relate to difficulties identifying the individual patient’s needs and the appropriate outcome measures for capturing the value of the health service received, as well as to the issue of whom to include in the outcome assessment and when. In the following section, we will discuss the value-based approach and the organizational boundaries that can threat the implementation. In addition, we will discuss and give examples of how the quality can be evaluated from a patient perspective and from an informal caregiver perspective.

## Discussion

### Organizational boundaries

Health services today are often fragmented [12, 13]; this is certainly true in Sweden, where several stakeholders are responsible for health-services delivery, mainly the municipality or the county councils [14]. Furthermore, since 2008, health-service providers have become more diversified; private health-service enterprises have proliferated in the wake of changed legislation [15]. Sweden also lacks an effective organizational framework for obtaining an overview of all health services coupled with responsibility for patients’ paths through the cycle of care. Our previous research has shown that patients overall do not experience safe transitions and that they consider personal agency necessary to ensuring the continuity of their care [16]. Consequently, given such fragmented care cycles and the lack of an authority with overarching responsibility, it is difficult to identify which part of the cycle of care should be evaluated in terms of value-based healthcare.

**Fig. 1:**
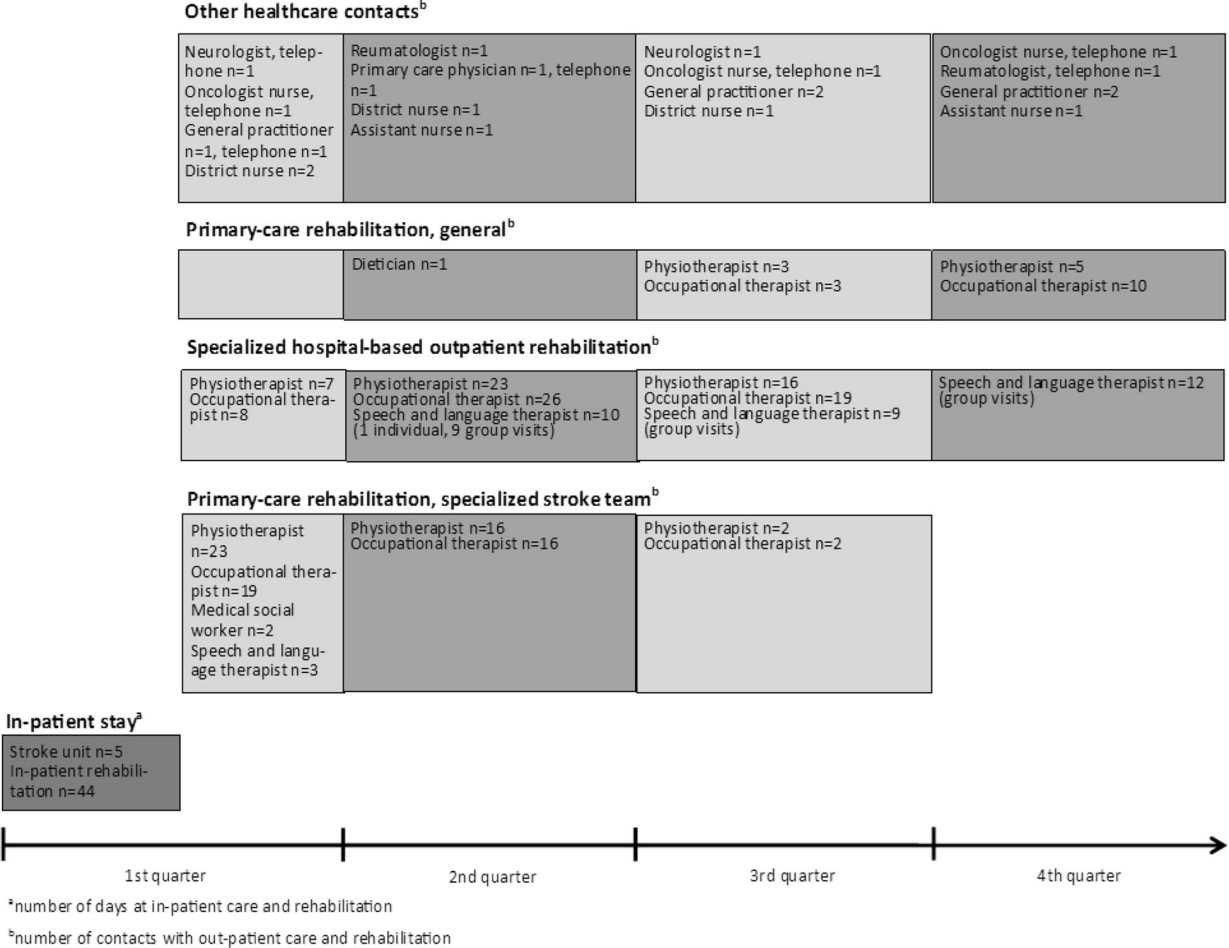
Erik’s use of health services (i.e. days spent in-patient care and contacts with out-patient care providers)

In contrast to the care cycle that follows surgery, as described by Porter [8], it is more challenging to define a cycle of care for a complex long-term health condition (e.g., after a stroke), when the need for health services related to the condition might be extended, even lifelong and may involve many different caregivers within different service levels and contexts. One of our case descriptions—Erik’s health services after his stroke (Table 1)—illustrates a common, albeit complex, use of health services during the first year after stroke. Figure 1 presents a summary of Erik’s use of health services (i.e., days spent in in-patient care and contacts with out-patient care providers). The beginning and the end of his cycle of care must be clearly defined, along with the outcomes related to each performance, in order to facilitate an evaluation of the quality of health services that aligns with value-based healthcare.

**Table 1:** Erik’s encounters with rehabilitation and other health services in the course of the first year after a stroke

Erik^a^ was 78 years old, lived with his wife and was independent in his activities of daily living when he had a stroke that caused moderate hemiparesis and aphasia. Erik received initial care in a stroke unit, where he stayed for 5 days; this period was followed by 44 days in a geriatric rehabilitation ward specializing in rehabilitation after stroke (Fig. 1). The same day he was discharged and returned home, a physiotherapist (PT) and an occupational therapist (OT) from a stroke team, organized by the primary-care centre, visited him at home for his first rehabilitation session. Erik faced activity limitations with regard to activities such as getting dressed, cutting his food and walking independently, and he was unable to climb stairs. His aphasia had improved, but he still had difficulties communicating. Initially, the PT and the OT visited him almost daily; thereafter these sessions were less frequent, but the rehabilitation sessions in Erik’s home with the stroke team continued throughout the first three quarters of the year. Supplementing his training with the stroke team, Erik’s wife and the home-help service daily encouraged his attempts to regain independence and to resume previously valued activities. In addition to the home rehabilitation, Erik also received rehabilitation treatment at the hospital’s geriatric out-patient clinic. The initial focus on physiotherapy and occupational therapy had by the end of the year changed to speech and language therapy. During the third quarter, when the home-based rehabilitation provided by the stroke team was completed, Erik began physiotherapy and occupational therapy at the primary-care centre. One year after the stroke, Erik had improved significantly but still had difficulty climbing stairs independently and speaking when he was tired or stressed. Apart from the rehabilitation provided at the stroke unit and at the in-patient rehabilitation ward, Erik received 235 rehabilitation sessions, provided by a PT and an OT, a medical social worker, speech and language therapists and a dietician. He also had other healthcare contacts, such as primary-care physicians and district nurses.	

^a^Erik is a pseudonym

### Evaluating the quality of health services

New measures of quality are needed that encourage coordination and the integration of health services across the cycle of care, creating incentives for providers to share responsibility for each patient’s health problem. Such measurements throughout the entire cycle of care can involve individual measures or a composite of several, and the health outcomes can be structured as health status achieved, outcomes related to the service itself (experience-based measure), and sustainability (how long the patient maintains health improvements) [8, 17]. These outcome measures are all relevant for improving health-services delivery, but how do the chosen outcome measures correspond to the value and expectations defined by patients? The description of Jenny (Table 2) illustrates that the patient’s goals may not coincide with goals set by the health professionals for the patient.

**Table 2:** Jenny’s needs and experiences in rehabilitation

Jenny^a^ is a 79-year-old woman who had a stroke four days ago. She lives alone and until now had been quite independent. Since the stroke, however, she has faced problems moving her right arm and hand. She can manage to get dressed, although it takes a long time, and she has to wear more casual clothes than she is used to because it is difficult to manage buttons and other closures on dressier or more formal clothing. She can eat with her left hand but needs help to cut the food. She has just arrived at the rehabilitation unit and has high expectations for her stay. She is very motivated and determined to return to her previous level of independence. After only a few days, Jenny is discharged and returns home, where she continues rehabilitation sessions with her primary-care stroke team. They visit her once a week, and after six weeks the rehabilitation ends. Jenny has improved her arm and hand function, and the stroke team considers the rehabilitation very successful, but Jenny has not returned to her previous level of independence. For example, though she had long taken great pleasure in cooking, Jenny now needs home-help service to deliver food boxes for her to heat in the microwave oven. Jenny is very disappointed and had hoped for a much more intensive rehabilitation.	

^a^Jenny is a pseudonym

Our previous research [18] has shown that outcome measures defined by health professionals do not always align with the outcomes most desired by and relevant for patients. These perspectives may differ, as shown by comparing problems after stroke reported in response to an open question (self-reported problems) with the problems captured by three standardized and commonly used assessment tools used for the same individuals at the same point in time. Items/domains in the assessment tools corresponded to only 15 of 24 categories of self-reported problems, and none of the assessment tool captured the most frequently reported problem (i.e., fatigue).

Care outcomes should be measured over the full cycle of care, be multidimensional and take into account complex conditions [9]. Porter et al. [19] suggest national surveillance to “ensure universal, consistent and fair measurement.” In reality, however, it might be difficult to attribute a beneficial outcome to a cycle of care. In Erik’s case, the significant improvement in activities of daily living might also be attributed to the home-help service or to his wife, who patiently supported him each time he struggled with his clothes or the cutlery in his ambition to regain his independence.

A prerequisite for evaluation in value-based healthcare constitutes registers that include data on patients’ healthcare contacts and healthcare activities. Sweden has a long tradition of quality healthcare registers, which are monitored and financed by an executive committee at the Swedish Association of Local Authorities and Regions (SKL) [20]. Currently, approximately 105 registers exist that provide data for management and research. The registers have previously collected data on processes (e.g., the number of people admitted to a stroke unit in the acute phase of stroke) and outcomes (e.g., mortality or complications) and nowadays the registers also increasingly include patient-reported outcome measures (PROMs) [21] and have started developing patient-reported experience measures (PREMs) [21]. A promising undertaking that will provide international standards for PROMs to be included in quality registers has begun at the initiative of an international consortium for health outcome measures (ICHOMs) [22].

The addition of PROMs to quality registers is a promising development with the potential to increase the presence of outcome measures that are important to patients. Nevertheless, the healthcare services must still consider the needs and goals of each individual because patient’s own goals may not be the same as those of the healthcare providers [23]. Ultimately, the measures should be the result of a shared decision-making between the patient and the healthcare provider, and when applicable, the family care-giver. Thus, flexible measurements are required that can capture the patient-centred perspective on an individual level, as illustrated by Jenny’s goal of regaining her cooking ability (Table 2), while still remaining possible to evaluate on an aggregated level. Some instruments are designed to capture the patient’s own healthcare goals and how well healthcare succeeds in meeting these goals (e.g., Goal Attainment Scaling [24, 25], the Canadian Occupational Performance Measure [26], and the Client-Centredness of Goal Setting scale [27]). But it is not sufficient to measure effectiveness alone in patient experiences of goal achievements; there is also a need to understand how well healthcare succeeds in terms of patients’ satisfaction with care and their health-related quality of life. An association between satisfaction with care and quality of life and adherence to treatment has been reported [21].

Including and using PREMs, which include both satisfaction with care and experiences of it, are essential to achieving patient participation in healthcare and healthcare management [24, 25]. We therefore argue that PREMs should be incorporated as a relevant outcome measure in managing long-term and complex conditions.

Quality registers and outcome measurements are assumed to support the use of evidence-based guidelines and enhance the quality. There has long been a routine for collecting and systematically analyzing data in order to understand the quality of care and identify areas for improvement [28]. However, studies have shown that measurements can facilitate improvements in care but neither the register itself nor reporting of data initiates quality improvements [29]. Thus, knowledge is needed about how data that capture health outcomes that matter to patients can feed quality-improvement initiatives in various contexts and how the involved stakeholders i.e. professionals co-operate.

### Informal caregivers’ perspective

The informal caregivers’ perspective should also be considered when choosing outcome measures in value-based healthcare. For patients living with long-term complex conditions, a large part of their health services might be performed by the patients themselves as self-management or by significant others, as illustrated by Erik’s case (Table 1). A systematic review of the situation of informal caregivers vis-à-vis patients who have had a stroke has shown increased and additional responsibilities for caregivers, leading to decreased time for leisure and paid work [30]. Being an informal caregiver thus may affect the caregiver’s own health [31, 32].

### Timing and commitment of measures from the patient’s perspective

The timing and extent of measures reported by patients that are required for measuring value must also be considered. In stroke rehabilitation, and as illustrated by Erik’s case (Table 1 and Fig. 1), several multi-professional teams (consisting of, e.g., physicians, nurses, physiotherapists, occupational therapists, medical social workers, speech therapists and dieticians) are involved from the first acute phase into the patient’s continued rehabilitation, resulting in a sometimes overlapping chain of care. Additionally, patients who have had a stroke often have a high prevalence of other chronic conditions that can involve healthcare professionals from several different organizations (Fig. 1). In the case of Jenny (Table 2), her perspective and valued goal was to our knowledge not reached when her rehabilitation was discontinued.

All this requires careful consideration when deciding what, when and how much should be measured. What should be measured is closely related to the goals set for the rehabilitation or care services. In our opinion, a truly value-based healthcare service should enable patients to participate in the goal setting for their own rehabilitation and to determine how goals are to be achieved, providers should not engage patients solely when it comes to reporting outcomes. When factors should be measured relates to the patient’s cycle of care and requires coordination among healthcare providers across both the continuum of care and different levels of healthcare. In addition, rehabilitation goals may take a long time to achieve; for example, outcomes related to adapting to a new life situation or to handling problems in daily life may take several months or years to attain. How much should be measured relates to the extent of reporting that is required to adequately measure all delivered health services without creating too great a burden for the patient.

## Conclusions

This paper has sought to critically reflect on value-based health services for patients with long-term conditions and complex needs and to point out some challenges. In value-based health services, relevant outcome measures with a linked reimbursement system are supposed to drive development towards higher quality. This shift has the potential to align healthcare-service delivery with patient-centred care in an attempt to take the patient’s perspective into account. We conclude, however, that the following issues remain to be considered before value-based health services can be implemented:

- In the development and establishment of PROMs and PREMs, these outcome measures must also be standardized to allow the evaluation of specific conditions at an aggregated level, but they must still be sensitive enough to capture each patient’s individual needs and goals.
- Strategies must be developed to manage the evaluation of which outcomes relate to which performance. This is especially apparent in fragmented healthcare systems and for patients with long-term—and multi-setting-care needs. Thus, healthcare systems that strive to establish value-based care must collaborate beyond organizational boundaries to create clear patient trajectories.
- The evaluation of outcome measures will not lead to quality improvements per se. Quality improvements require, among other things, knowledge about how data that capture health outcomes that matter to patients can feed quality-improvement initiatives in various contexts. Thus there are challenges that need to be addressed before value-based health services are likely to drive quality improvement for people with complex long-term conditions.

## Abbreviations

ICHOMs: International consortium for health outcome measures
PREMs: Patient-reported experience measures
PROMs: Patient-reported outcome measures

## References

[1] Mead N, Bower P (2000). Patient-centredness: a conceptual framework and review of the empirical literature. Soc Sci Med.

[2] Ekman I, Swedberg K, Taft C, Lindseth A, Norberg A, Brink E, et al. Person-centered care--ready for prime time. Eur J Cardiovasc Nurs. 2011;10(4):248–51. 10.1016/j.ejcnurse.2011.06.008 21764386

[3] Institute of Medicine (IOM) (2001). Crossing the Quality Chasm. Crossing the Quality Chasm: A New Health System for the 21st Century.

[4] Swedish Health and Medical Service Act. The patient law (patientlagen). 2014:821.

[5] Docteur E, Coulter A (2012). Patient-Centeredness in Sweden’s Health System An assessment and six steps for progress. The Swedish Agency for Health and Care Services Analysis.

[6] Vårdanalys (The Swedish Agency for Health and Care Services Analysis) (2014). VIP i vården?—Om utmaningar i vården av personer med kronisk sjukdom (VIP in healthcare?—challenges in care of the persons with chronic disease).

[7] Vårdanalys (The Swedish Agency for Health and Care Services Analysis) (2014). Vården ur patienternas perspektiv—jämförelser mellan Sverige och 10 andra länder (The care from patient perspective—comparsion between Sweden and other countries).

[8] Porter ME (2008). Value-based health care delivery. Annals Surg.

[9] Porter ME, Pabo EA, Lee TH (2013). Redesigning Primary Care: A Strategic Vision To Improve Value By Organizing Around Patients’ Needs. Health Aff.

[10] Porter ME (2010). What is value in health care. N Engl J Med.

[11] Little P, Everitt H, Williamson I, Warner G, Moore M, Gould C, Ferrier K, Payne S (2001). Preferences of patients for patient centred approach to consultation in primary care: observational study. Br Med J (Clin Res Ed).

[12] Institute of Medicine (IOM) (2006). Hospital-Based Emergency Care: At the Breaking Point.

[13] Statens offentliga utredingar (SOU). Swedish Government Official Reports 2016:6. Effective care (Effektiv vård). Stockholm: Swedish Government; 2016.

[14] Wadmann S, Strandberg-Larsen M, Vrangbaek K (2009). Coordination between primary and secondary healthcare in Denmark and Sweden. Int J Integr Care.

[15] SFS (Svensk författningssamling) (Swedish Statue). Law on system of choice (Lag om valfriehetssystem). 2008:962.

[16] Flink M, Hesselink G, Pijnenborg L, Wollersheim H, Vernooij-Dassen M, Dudzik-Urbaniak E, Orrego C, Toccafondi G, Schoonhoven L, Gademan P (2012). The key actor—a qualitative study of patient participation in the handover process in Europe. BMJ Qual Saf.

[17] McHugh M, Joshi M (2010). Improving evaluations of value-based purchasing programs. BMC Health Serv Res.

[18] Tistad M, Ytterberg C, Tham K, von Koch L (2012). Poor concurrence between disabilities as described by patients and established assessment tools three months after stroke: A mixed methods approach. J Neurol Sci.

[19] Porter ME (2009). A strategy for health care reform—toward a value-based system. N Engl J Med.

[20] Swedish Association of Local Authorities and Regions. http://skl.se/tjanster/englishpages.411.html. Accessed 20 Nov 2016.

[21] PROMcenter (Patient reported outcome measurements) (2013). The correlations between satisfaction with, and experience of care and patient-reported outcomes—a literature review. Sambanden mellan tillfredssställelse med och upplevelse av vården och patientrapporterat utfall—en litteraturöversikt.

[22] The International Consortium for Health Outcomes Measurement (ICHOM). http://www.ichon.org. Accessed 20 Nov 2016.

[23] Di Blasi Z, Harkness E, Ernst E, Georgiou A, Kleijnen J (2001). Influence of context effects of health outcomes: a systematic review. Lancet.

[24] Hurn J, Kneebone I, Cropley M (2006). Goal setting as an outcome measure: A systematic review. Clin Rehabil.

[25] Kiresuk TJ, Sherman RE (1968). Goal attainment scaling: a general method of evaluating comprehensive mental health programmes. J Commun Ment Health.

[26] Law M, Baptiste S, McColl M, Opzoomer A, Polatajko H, Pollock N (1990). The Canadian Occupational Performance Measure: an outcome measure for occupational therapy. Can J Occup Ther.

[27] Doig E, Prescott S, Fleming J, Cornwell P, Kuipers P (2015). Development and construct validation of the Client-Centredness of Goal Setting (C-COGS) scale. Scand J Occup Ther.

[28] Batalden PB, Davidoff F (2007). What is “quality improvement” and how can it transform healthcare?. Qual Saf Health Care.

[29] Eldh AC, Fredriksson M, Halford C, Wallin L, Dahlström T, Vengberg S, Winblad U. Facilitators and barriers to applying a national quality registry for quality improvement in stroke care. BMC Health Serv Res. 2014;14:354–4. 10.1186/1472-6963-14-354 PMC4153899 25158882

[30] Pellerin C, Rochette A, Racine E (2011). Social participation of relatives post-stroke: the role of rehabilitation and related ethical issues. Disabil Rehabil.

[31] Bergström AL, von Koch L, Andersson M, Tham K, Eriksson G (2015). Participation in everyday life and life satisfaction in persons with stroke and their caregivers 3–6 months after onset. J Rehabil Med.

[32] Bertilsson A-S, von Koch L, Tham K, Johansson U (2015). Client-centred ADL intervention after stroke: Significant others’ experiences. Scand J Occup Ther.

